# The Characterization of Arabidopsis *mterf6* Mutants Reveals a New Role for mTERF6 in Tolerance to Abiotic Stress

**DOI:** 10.3390/ijms19082388

**Published:** 2018-08-14

**Authors:** Pedro Robles, Sergio Navarro-Cartagena, Almudena Ferrández-Ayela, Eva Núñez-Delegido, Víctor Quesada

**Affiliations:** Instituto de Bioingeniería, Universidad Miguel Hernández, Campus de Elche, 03202 Elche, Spain; probles@umh.es (P.R.); s.navarro@umh.es (S.N.-C.); sikalea@hotmail.com (A.F.-A.); eva.nunez@goumh.umh.es (E.N.-D.)

**Keywords:** Arabidopsis, mitochondrial transcription termination factor (mTERF), salt stress, abiotic stresses, abscisic acid (ABA), organellar gene expression (OGE)

## Abstract

Exposure of plants to abiotic stresses, such as salinity, cold, heat, or drought, affects their growth and development, and can significantly reduce their productivity. Plants have developed adaptive strategies to deal with situations of abiotic stresses with guarantees of success, which have favoured the expansion and functional diversification of different gene families. The family of mitochondrial transcription termination factors (mTERFs), first identified in animals and more recently in plants, is likely a good example of this. In plants, mTERFs are located in chloroplasts and/or mitochondria, participate in the control of organellar gene expression (OGE), and, compared with animals, the mTERF family is expanded. Furthermore, the mutations in some of the hitherto characterised plant mTERFs result in altered responses to salt, high light, heat, or osmotic stress, which suggests a role for these genes in plant adaptation and tolerance to adverse environmental conditions. In this work, we investigated the effect of impaired mTERF6 function on the tolerance of Arabidopsis to salt, osmotic and moderate heat stresses, and on the response to the abscisic acid (ABA) hormone, required for plants to adapt to abiotic stresses. We found that the strong loss-of-function *mterf6-2* and *mterf6-5* mutants, mainly the former, were hypersensitive to NaCl, mannitol, and ABA during germination and seedling establishment. Additionally, *mterf6-5* exhibited a higher sensitivity to moderate heat stress and a lower response to NaCl and ABA later in development. Our computational analysis revealed considerable changes in the *mTERF6* transcript levels in plants exposed to different abiotic stresses. Together, our results pinpoint a function for Arabidopsis mTERF6 in the tolerance to adverse environmental conditions, and highlight the importance of plant mTERFs, and hence of OGE homeostasis, for proper acclimation to abiotic stress.

## 1. Introduction

The increased salt content in arable soils severely compromises plant growth and productivity. This is due to osmotic stress, which promotes water loss and hinders its uptake by plant roots, and to ionic stress (Na^+^ and Cl^−^ in most cases), which generates toxicity and hinders the recruitment of other ions [1]. The development of new varieties of more halotolerant crop plants requires unravelling the genetic and molecular mechanisms that underlie tolerance to salinity. It has been proposed that chloroplasts could act as sensors capable of sensing environmental stress, and, by retrograde signalling (from the chloroplast to the nucleus), could coordinate the expression of nuclear genes that allow plants to adapt to stress [2]. In line with this, Leister et al. [3] have reported that perturbed organellar gene expression (OGE) homeostasis activates the acclimation and tolerance responses of plants, likely through retrograde communication. Notwithstanding, information about chloroplasts involvement in the response to abiotic stress in general, and to salinity in particular, is still scarce. We initiated a bioinformatics and reverse genetics approach in the plant model system *Arabidopsis thaliana* to identify novel functions involved in the control of gene expression in chloroplasts. We previously identified and characterised two genes, *MDA1* [4] and *mTERF9* [5], not previously described, which belong to the family of mitochondrial transcription termination factors (mTERFs) [6]. The analysis of the *mda1* and *mterf9* mutants revealed a connection between chloroplast function and the response to salt stress and ABA in Arabidopsis [4,5]. For other Arabidopsis *mTERF* genes besides *MDA1* and *mTERF9*, a role in acquiring tolerance to salinity (*mTERF10* and *mTERF11*) [7], heat (*SHOT1* (*SUPPRESSOR OF HOT1-4 1*)) [8] or high light (*SOLDAT10* (*SINGLET OXYGEN-LINKED DEATH ACTIVATOR10*) [9]) has also been reported. Accordingly, *mda1* and *mterf9* are less sensitive to NaCl than the wild type [4,5], *mterf10* and *mterf11* are salt-hypersensitive [7], whereas *shot1* [8] and *soldat10* [9] show enhanced heat tolerance and constitutive acclimation to light, respectively. In addition to Arabidopsis, the *stm6* mutant (*state transition mutant6*) of the green algae *Chlamydomonas reindhardtii* affected in the *MOC1* (*mterf-like gene of Chlamydomonas1*) gene is light sensitive [10]. Along this line, an emerging role for some *mTERF* genes in the response, tolerance, and/or acclimation of plants to different abiotic stress conditions has been recently proposed, which might, at least in part, explain the expansion and diversification of the plant mTERF family compared with that of animals [11]. 

mTERF proteins share the presence of a variable number of repeats of a motif called mTERF of about 30 amino acids. In vertebrates, four subfamilies have been identified (MTERF1-4), in which the MTERF1 protein is the first to be characterised [12]. However, plant genomes, especially those from higher plants, contain a larger number of *mTERF* genes than animal genomes [13]. In metazoans, mTERF proteins participate in the control of mitochondrial transcription, and are required for both its initiation and termination [14]. In plants, several molecular functions have been proposed for some of the *mTERF* genes hitherto characterised, all of which are related to the posttranscriptional regulation of chloroplasts and/or mitochondria gene expression. Accordingly, Arabidopsis mTERF15 [15] and maize Zm-mTERF4 [16] are involved in intron splicing in mitochondria, Arabidopsis BELAYA SMERT/RUGOSA2 [17,18] is required for intron splicing in plastids, and *Chlamydomonas reindhardtii* MOC1 promotes the termination of antisense mitochondrial transcription [19]. The Arabidopsis mTERF6 protein, dually targeted to chloroplasts and mitochondria, is involved in the maturation of the chloroplast isoleucine tRNA (*trnI.2*) gene and the aminoacylation of tRNA for isoleucine [20,21].

We previously identified and morphologically characterized a new mutant allele of the Arabidopsis AT4G38160 (*mTERF6*) gene which we dubbed *mterf6-5* after finding it to be allelic of the previously described *mterf6-2* mutant [20,22]. *mterf6-2* and *mterf6-5* are insertional alleles of the SAIL and SALK collection of T-DNA lines (SAIL_360_H09 and SALK_116335 respectively). *mTERF6* transcripts were undetectable in *mterf6-2* plants [20], and significantly reduced in the *mterf6-5* mutant [22]. This caused a substantial delay in plant growth, smaller size than the wild type, and loss of pigmentation in cotyledons, leaves, stems, sepals, and fruits in both mutants. In our growth conditions, these phenotypic traits were much more marked in *mterf6-2* than in *mterf6-5* [22]. Altogether, the data suggest that *mterf6-2* and *mterf6-5* are null and strong hypomorphic alleles respectively, of the *mTERF6* gene [20,22]. Furthermore, the *mterf6-5* mutation enhanced the leaf polarity defects of the *asymmetric leaves1* mutant, and revealed a role for the *mTERF6* gene in adaxial-abaxial leaf patterning [22]. Nevertheless, whether this gene plays a role in tolerance to abiotic stress as reported for other *mTERF* genes remains to be evaluated. To investigate this, we report herein the study of the response of the wild-type Col-0 and the strong loss-of-function *mterf6-2* and *mterf6-5* alleles to the ionic and osmotic stresses caused by the presence of high concentrations of NaCl or mannitol in culture media, respectively. We also evaluated the sensitivity of *mterf6-2* and *mterf6-5* to the abscisic acid (ABA) hormone, involved in plant adaptations to environmental stress. Our results revealed an altered response of the *mterf6* mutants to the stress conditions assayed, which is consistent with the substantial changes in *mTERF6* expression we found in silico after exposing the wild-type to different abiotic stresses. 

## 2. Results

### 2.1. The mterf6-2 and mterf6-5 Mutants Are Hypersensitive to NaCl and Mannitol

**Figure 1:**
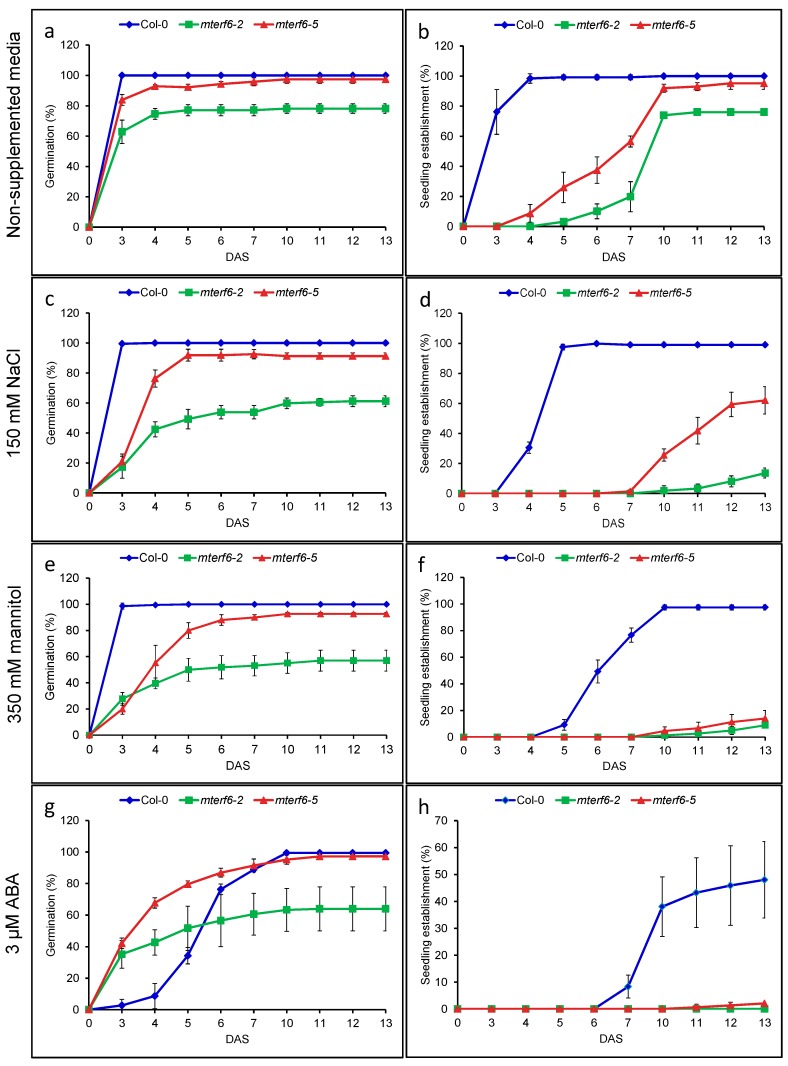
Effects of NaCl, mannitol and ABA on germination and seedling establishment in the wild-type Col-0 and the *mterf6-2* and *mterf6-5* mutants. Each value corresponds to the mean ± standard deviation (SD) of the percentage of germination (**a**,**c**,**e**,**g**) and seedling establishment (**b**,**d**,**f**,**h**) in the growth media either without supplementation (**a**,**b**) or supplemented with 150 mM of NaCl (**c**,**d**), 350 mM of mannitol (**e**,**f**) or 3 µM of ABA (**g**,**h**) of four replicates of at least 50 seeds each per genotype. DAS: days after stratification.

In order to assess whether the *mterf6-5* mutant that we previously identified [22] exhibited altered sensitivity to abiotic stresses, we first analysed its sensitivity, and that of the wild-type Col-0, to the ionic stress produced by NaCl and osmotic stress due to mannitol. We also included the *mterf6-2* mutant, allelic of *mterf6-5,* in the analysis (see above). For this purpose, we first examined the ability of *mterf6-2, mterf6-5* and Col-0 seeds to germinate and to form fully expanded green cotyledons (seedling establishment) in the first 2 weeks after seed stratification in the presence of 0, 150, or 200 mM of NaCl or 350 mM of mannitol. In the non-supplemented culture medium, mutants *mterf6-2* and *mterf6-5* respectively yielded, to some extent, lower and similar seed germination ratios than Col-0 (we considered germinated those seeds in which radicle emergence through the seed coat was observed) (Figure 1a). The supplementation of growth medium with NaCl (150 mM) or mannitol (350 mM) did not affect wild-type seed germination, but lowered the *mterf6-2* and *mterf6-5* germination rates, especially those of the former, and the effect was more pronounced from 1 to 5 DAS (days after stratification; Figure 1c,e). 

Consistent with the stunted growth of the *mterf6-2* and *mterf6-5* individuals [20,22], the seedling establishment of both mutants was delayed compared with Col-0 (e.g., at 6 DAS in the MS control medium, 10%, 37%, and 99% of the *mterf6-2, mterf6-5* and Col-0 seeds yielded seedlings with fully expanded green cotyledons, respectively (Figure 1b)). However, at 10 DAS, 93% and 100% of seedling establishments were achieved for the *mterf6-5* and the wild type, respectively, whereas *mterf6-2* reached a maximum value of 77% at 11 DAS (Figure 1b). The *mterf6-2* and *mterf6-5* seeds yielded substantially lower seedling establishment rates than those of Col-0 in the presence of 150 mM of NaCl or 350 mM of mannitol (Figure 1d,f). Accordingly, the presence of the *mterf6-2* and *mterf6*-5 seedlings with green expanded cotyledons could be scored only from 10 DAS in the presence of NaCl or mannitol, while the seedling establishment for Col-0 was observed from 4–5 DAS under the same conditions (Figure 1d,f). Notwithstanding, the *mterf6-2* mutant was more sensitive than *mterf6-5* to NaCl. In line with this, the maximum seedling establishment values for *mterf6-2* and *mterf6-5* in 150 mM NaCl were 14% and 62%, respectively, which were reached at 13 DAS, whereas Col-0 yielded ~100% (Figure 1d). However, a similar strong hypersensitive response to mannitol was found for both mutants throughout the study period (Figure 1f). 

We also investigated the response of Col-0 and *mterf6-5* to a higher salt concentration by supplementing the culture medium with 200 mM of NaCl. We found that this condition significantly delayed mutant germination (e.g., at 5 and 10 DAS, 98% and 99% of the wild type and 12% and 80% of the *mterf6-5* seeds germinated, respectively, in 200 mM of NaCl; Table S1), and completely abolished the Col-0 and *mterf6-5* seedling establishments, as we were unable to identify any individual that displayed green expanded cotyledons. 

Taken together, our results revealed enhanced sensitivity to salt and osmotic stress during germination, and mainly in the cotyledon greening stage, for the studied *mterf6* mutants.

We evaluated the response of *mterf6-5* to salinity by exposing plants to stress after germination and seedling establishment. To this end, 5 DAS wild-type and mutant seedlings were transferred from the non-supplemented medium to the media supplemented with NaCl (125 or 150 mM), and root length was determined 8 days after transfer (13 DAS; see Materials and Methods; Table S2). The *mterf6-5* plants were significantly less sensitive than the wild-type ones to the inhibition of root growth caused by the presence of either 125 mM of NaCl or 150 mM of NaCl (Table 1; Table S2).

To study whether a low *mTERF6* expression altered tolerance to moderate heat stress, the wild-type Col-0 and *mterf6-5* mutant seedlings were exposed 13 days to a higher (28 °C) than normal culture temperature (20 °C). We also compared the response of *mterf6-5* with that of *mterf* mutants *mda1-1* and *mterf9*. The *mterf6-5* mutant was hypersensitive to heat stress because paleness markedly increased and seedling growth was severely impaired, and even arrested, when grown at 28 °C (Figure S1). In contrast, the growth of *mda1-1* and *mterf9* was enhanced at 28 °C, but to a lesser extent than in Col-0 (Figure S1).

### 2.2. Knock-Down of mTERF6 Alters the Response to ABA

The abscisic acid (ABA) hormone plays a fundamental role in seed germination and in the responses of plants to abiotic stresses [23]. The Arabidopsis mutants deficient in ABA signalling or biosynthesis also exhibited enhanced tolerance to salt stress [24,25,26]. Therefore, given the enhanced sensitivity of *mterf6-2* and *mterf6-5* to salt and osmotic stress, we investigated whether they also exhibited an altered response to ABA by growing the *mterf6* mutant and Col-0 seedlings in the presence of ABA. As shown in Figure 1a,g, 3 µM of ABA substantially delayed *mterf6-2, mterf6-5* and Col-0 germination, but from 3 to 5 DAS both mutant seeds exhibited higher levels of radicle emergence through the seed coat than those of the wild-type. However, when the *mterf6-5* and Col-0 individuals were grown on 6 µM of ABA, seed germination was greater in *mterf6-5* than in Col-0 only at 5 DAS, but both genotypes yielded very low germination values (6% and 3%, respectively; Table S3). In contrast, we found that *mterf6-5* was hypersensitive to ABA from 6–13 DAS. Accordingly at 6, 7, 10, and 13 DAS, 44%, 62%, 99%, and 100% of the Col-0 seeds, and 22%, 36%, 60%, and 82% of the *mterf6-5* seeds germinated, respectively (Table S3). 

As regards seedling establishment, exposure to 3 µM ABA considerably reduced it in Col-0 (e.g., up to 48% of the wild-type seedlings under the control condition at 13 DAS), and completely abolished it in *mterf6-2*, while only 2% was found for *mterf6-5* (Figure 1h). When grown on 6 µM ABA, 18% and 42% of the Col-0 seeds yielded seedlings with green expanded cotyledons at 10 and 13 DAS, respectively. As expected, no *mterf6-5* seedlings showing green expanded cotyledons were found from 3 to 13 DAS (Table S3). 

We allowed the Col-0 and *mterf6-5* seedlings to grow on the ABA-supplemented medium. At 17 DAS, 6.4% and 1.4% of the mutant seeds (*n* = 150) yielded individuals that displayed two very tiny leaves in 3 and 6 µM of ABA, respectively. In contrast, 27.7% and 16.2% of the Col-0 seedlings (*n* = 150) displayed two small leaves in 3 and 6 µM of ABA, respectively. 

Taken together, these results indicate that the *mterf6-2* and *mterf6-5* mutants are hypersensitive to ABA principally during seedling establishment.

As we did for NaCl (see Section 2.1), we also investigated the sensitivity of *mterf6-5* to ABA after germination and seedling establishment. To this end, 5 DAS wild-type and mutant plants were transferred from the non-supplemented medium to the media supplemented with ABA (5 or 10 µM). Root length was determined 8 days after transfer (13 DAS; Table S2). As well as for NaCl, the root growth of the *mterf6-5* individuals was significantly more tolerant than that of the Col-0 plants to 5 µM of ABA, whereas inhibition of root length only slightly decreased in 10 µM of ABA (Table 1). 

### 2.3. The Expression of the mTERF6 Gene Changes in Response to Abiotic Stresses

Given the altered sensitivity of the *mterf6* mutants to NaCl, mannitol and ABA, we decided to perform an in silico analysis of the expression of the *mTERF6* gene in response to different abiotic stress conditions. Hence we studied the stress-induced changes in the transcript levels of *mTERF6* with the Arabidopsis AtGenExpress Visualization Tool ([27]; available online: http://weigelworld.org/resources.html) in the roots and aerial parts of the Col-0 seedlings under NaCl, osmotic and drought stresses. The expression values were plotted over time (0, 0.5, 1, 3, 6, 12, and 24 h after treatment started) to obtain a graphical representation of the response of *mTERF6* to these conditions (Figure S2). Compared with the untreated plants, *mTERF6* expression was down-regulated in the green parts of seedlings after 3 h of NaCl (150 mM), mannitol (300 mM) and drought treatments, and mostly in the presence of NaCl and mannitol from 6 to 24 h. This repression peaked 24 h after treatment when the *mTERF6* transcript levels lowered to 16% and 42% of the control plants in response to mannitol and NaCl, respectively (Figure S2a). As regards roots, *mTERF6* expression was down-regulated by salt stress from 1 to 24 h after treatment started. The difference to the control plants was maximum at 6 h (38.6% of the control plants), whereas mannitol slightly increased the *mTERF6* transcript levels at 3 h (28% more than in the control plants), but lowered them from 6 to 24 h, especially at 6 h (63% of the control plants) (Figure S2b). Drought reduced *mTERF6* expression at 1 and 6 h after exposure (77% and 74% of the control plants), but no appreciable differences were found for the remaining time points. As regards the effect of ABA, *mTERF6* expression was down-regulated to 53.4% of the control plants by 10 µM ABA after 3 h, but no noticeable differences were found after 0.5 and 1 h. We also investigated the transcript levels of *mTERF6* using the online data from the At-TAX Arabidopsis whole genome tilling array [28]. Consistently with the AtGenExpress results, we found that the 12-h exposure of the 10-day-old Col-0 seedlings to 200 mM of NaCl, 300 mM of mannitol or 100 µM of ABA markedly reduced *mTERF6* transcript abundance to 30.4%, 45.5% and 45.2% of those of the untreated seedlings, respectively. Slighter differences between the treated and untreated plants were detected after 1 h of exposure under the same conditions.

We experimentally tested by qRT-PCR whether *mTERF6* expression may change in response to NaCl. To this end, RNA was extracted from Col-0 seedlings collected 10 DAS and grown in GM medium supplemented with 100 mM NaCl or in non-supplemented medium. The RNA was retro-transcribed and the cDNAs analyzed by qPCR. Though this condition was different from those used by the Arabidopsis AtGenExpress consortium (see above; [27]), we previously found that it delayed Col-0 growth [4]. We included as a positive control the *RD29A* gene which is induced by salinity [29]. In response to this moderate salt stress, *RD29A* was significantly upregulated (1.7 ± 0.3; *p* = 10^−3^) whereas *mTERF6* was slightly downregulated (0.8 ± 0.4; *p* = 0.2).

## 3. Discussion

In this work, we analysed the response of the *mterf6-2* and *mterf6-5* mutants to different abiotic stresses during germination, seedling establishment and for *mterf6-5* later in development. We found that the *mterf6* mutants displayed altered sensitivity to salt, osmotic stress, ABA, and moderate heat stress. Unlike the results obtained with other *mterf*-deficient mutants, such as *mda1* and *mterf9,* which are more insensitive than the wild type to such stresses [4,5], *mterf6-2* and *mterf6-5* were hypersensitive to the inhibition exerted on germination and seedling establishment by high concentrations of NaCl, mannitol, or ABA. *mterf6-2* was always more sensitive than *mterf6-5* to the different abiotic stress conditions studied, which is consistent with its more severe morphological phenotype [22]. The susceptibility of *mterf6* mutants to NaCl was similar to that of *mterf10* and *mterf11* [7], but unlike these mutants, which were as sensitive as the wild type was to ABA, *mterf6-2* and *mterf6-5* were also hypersensitive to this hormone, mainly during seedling establishment. 

In line with this, the knock-down of *mTERF6* also reduced seedling tolerance to moderate heat stress and led to impaired growth and development, whereas *mda1-1* and *mterf9* (this work), and *mterf10* and *mterf11* [7], did not show a significantly different response from that of the wild type under this condition. mTERF6 seemed to play a different role further in vegetative development because the deficient *mTERF6* function significantly reduced the sensitivity of roots to the presence of NaCl or ABA in the growth medium. A different susceptibility to salt and ABA during germination and vegetative growth has been previously reported for mutants *mda1* and *mterf9* [4,5]. 

We extracted the *mTERF6* transcript levels from AtGenExpress [27] by selecting “AtGE Abiostress” as a data source. Consistent with altered tolerance to abiotic stresses, we found that *mTERF6* expression was markedly down-regulated in response to salt, osmotic stress (mannitol) and drought, especially after prolonged exposure (12–24 h) to 150 mM NaCl and 300 mM mannitol. Interestingly, ABA treatment also repressed *mTERF6* expression. We experimentally tested by qRT-PCR *mTERF6* expression in 10 DAS plants grown in mild salt stress conditions (100 mM NaCl), and found that it was slightly but not significantly downregulated, which is likely due to the different stress conditions used to study *mTERF6* expression. Together, our results suggest that the altered tolerance of *mterf6-2* and *mterf6-5* to the tested abiotic stresses could be attributed to its different sensitivity to ABA compared with the wild type, because this hormone plays a fundamental role in plants’ response and adaptation to abiotic stress conditions. The involvement of *mTERF6*, *MDA1* (affected in the *mTERF5* gene), and *mTERF9* (the *mda1* and *mterf9* mutants are less sensitive to ABA than the wild type) [4,5], and possibly of *mTERF10* (since a modest overexpression of this gene leads to enhanced germination and growth in the presence of ABA) [7] in abiotic stress tolerance could take place through ABA signalling. Accordingly, several pieces of experimental evidence indicate a role for ABA in plastid-to-nucleus signalling (reviewed in [3]). Therefore, the impaired plastid gene expression may be due to a defective mTERF function perturbing the retrograde communication (from plastids to the nucleus) mediated by ABA under salt or other abiotic stress conditions. As a result, this would alter nuclear gene expression, and hence, tolerance to these environmental conditions. Similarly, Leister and Kleine [21] found that levels of the nuclear transcripts, which encode the chloroplast proteins involved in organellar gene expression (OGE), were affected in the weak *mterf6-1* mutant. Notwithstanding, while some mTERF proteins (e.g., mTERF5, mTERF9 and mTERF10) would negatively modulate Arabidopsis salt tolerance as their down-regulation diminishes sensitivity to ABA and abiotic stresses, mTERF6 would play the opposite role by promoting such tolerance, at least during germination and seedling establishment. Consequently, it could be hypothesised that the outcome of the activity of different mTERF proteins, which act during germination and early vegetative development, might contribute to responses to abiotic stress in these developmental stages. The mTERF6 function in abiotic responses might be conserved in other plant species because the expression of the maize *mTERF12* gene, the orthologue of Arabidopsis mTERF6, is substantially altered after NaCl or ABA treatments [30]. Interestingly, the transcript levels of other maize *mTERF* genes also change after exposing maize plants to light/dark treatments, salt, ABA or 1-Naphthaleneacetic acid exposure [30]. The altered levels of the *mTERF6* transcripts after abiotic stress treatments found in silico might be interpreted as being necessary for plants to adapt to adverse environmental conditions. Nevertheless given currently available molecular information, we cannot rule out the notion that changes in the expression of *mTERF6* and other *mTERF* genes under different abiotic stress conditions might result from the perturbation of certain biological processes. Chloroplast homeostasis is likely to be one of these processes altered in *mterf*-deficient mutants, because all the mTERFs involved in the response to salt stresses described to date are targeted to chloroplasts; they also belong to the “chloroplast cluster” (mTERF5, mTERF6 and mTERF9) or to the “chloroplast associated-cluster” (mTERF10 and mTERF11) of proteins by functioning in organelle gene expression, embryogenesis, gene expression, and/or protein catabolism in plants [13]. The altered OGE, and hence chloroplast homeostasis, would account for the delayed growth and greening of the cotyledons of the *mterf6* individuals in relation to Col-0, even in the absence of abiotic stress. However, differences with Col-0 considerably increased when the *mterf6* mutants were exposed to salt, mannitol, or ABA, which indicates that *mterf6-2* and *mterf6-5* sensitivity to abiotic stresses cannot be attributed solely to its defective growth.

The involvement of the mTERF family of genes in the acclimation and tolerance of plants to different abiotic stresses conditions [11,14] is further supported by recent findings in cotton (*Gossypium barbadense*). Accordingly, multiple stress responsive genes have been identified in *G. barbadense* using a normalised cDNA library, constructed after exposure to various abiotic (heat, cold, salt, drought, potassium, and phosphorous deficit) and biotic (*Verticillium dahlia* infection) stress conditions [31]. Remarkably, the mRNAs of 464 transcription factors (TF) have been enriched in this library, and mTERFs are one of the most abundant TF families to have been identified (3.7%) [31]. 

## 4. Materials and Methods

### 4.1. Plant Material and Growth Conditions

Plant cultures and crosses were performed as previously described [4]. The seeds of the *Arabidopsis thaliana* (L.) Heynh. wild-type (WT) accession Columbia-0 (Col-0) were obtained from the Nottingham Arabidopsis Stock Centre (NASC). Seeds of the transferred DNA (T-DNA) insertion lines SAIL_360_H09 (*mterf6-2*), SALK_116335 (*mterf6-5*), SALK_597243 (*mda1-1*) and WiscDsLox474E07 (*mterf9*) were provided by the NASC and are described on the SIGnAL website (available online: http://signal.salk.edu). 

### 4.2. Germination and Growth Sensitivity Assays

For the germination assays, sowings were carried out as described in [4] on Petri dishes filled with GM agar medium (Murashige and Skoog (MS) medium containing 1% sucrose), supplemented with NaCl (150 and 200 mM), mannitol (350 mM) or ABA (3 and 6 µM). The seeds in which radicle emergence was observed were considered to be germinated, whereas seedling establishment was determined as those seedlings that exhibited green and fully expanded cotyledons. Seed germination and seedling establishment were scored from 1 to 13 DAS or from 1 to 24 DAS on Petri dishes, kept at 20 ± 1 °C with 72 µmol·m^−2^·s^−1^ of continuous light. 

To determine the salt and ABA responses during vegetative growth after seedling establishment, seeds were sown on non-supplemented GM agar medium, and seedlings were transferred on 5 DAS to new Petri dishes supplemented with NaCl (125 or 150 mM) or ABA (5 or 10 µM), and vertically grown. Plant root length was determined after 8 days of NaCl or ABA treatment to evaluate their tolerance to these stress conditions by referring the values to those of the individuals transferred to the control (non-supplemented) media. 

For the heat-sensitivity assays, the Col-0, *mda1-1*, *mterf9,* and *mterf6-5* plants were grown on Petri dishes at 28 ± 1 °C and 20 ± 1 °C for 14 DAS.

### 4.3. Quantitative RT-PCR (qRT-PCR)

Total RNA was extracted from 80 mg 10 DAS wild-type Col-0 plants grown in the presence or absence of 100 mM NaCl in the GM agar medium. The RNA was resuspended in 40 μL of RNase-free water and DNA removed using the TURBO DNAfree kit (Invitrogen, Waltham, MA, USA) following the manufacturer’s instructions. The cDNA preparations and qPCR amplifications were carried out in an ABI PRISM 7000 Sequence Detection System (Applied Biosystems, Waltham, MA, USA) as described in [4] using the oligonucleotides listed on Table S4. Each reaction mix of 20-μL contained 7.5 μL of the SYBR-Green/ROX qPCR Master Kit (Fermentas, Waltham, MA, USA), 0.4 μM of primers, and 1 μL of the cDNA solution. Relative quantification of gene expression data was performed by the 2^−ΔΔ*C*t^ method as described in [4]. Each reaction was done in three replicates, and three different biological replicates were used. The expression levels were normalised to the CT values obtained for the housekeeping *ACTIN2* gene [32], and a Mann–Whitney U-test was applied to the relative expression data obtained. 

### 4.4. Computational Analyses

The expression responses of the *mTERF6* gene under ABA, salt, osmotic, and drought stress were obtained from the AtGenExpress Visualization Tool (available online: http://jsp.weigelworld.org/expviz/expviz.jsp) [27] by selecting the “AtGE Abiostress” as the data source and mean-normalised values. The *mTERF6* expression in response to ABA was also visualised by extracting the tilling array data from TileViz (available online: http://jsp.weigelworld.org/tileviz/tileviz.jsp) [28] by selecting the “Abiotic Stress Dataset” and the mean-normalised values. 

## 5. Conclusions

In summary, the results reported herein reveal a new function for the *mTERF6* gene related to the emerging roles that have been recently proposed for the mTERF family in plants’ response and adaptation to different environmental stress conditions. In the plant *mterf* mutants characterised to date which have exhibited altered sensitivity to abiotic stresses, the affected mTERF proteins are involved in OGE [11,13,14]. Hence, this pinpoints an important function for OGE and plastid homeostasis, likely by acting throughout retrograde signalling, in tolerance to adverse environmental conditions, as recently proposed [3]. Further molecular research on the effect of abiotic stresses on the mTERF6 function, and by extension on the remaining mTERFs, is required to shed more light on the contribution of this scarcely known family of genes for plants to cope with abiotic stresses. 

## Figures and Tables

**Table 1:** Tolerance of the *mterf6-5* mutant to NaCl and abscisic acid (ABA).

Genotype	Inhibition of Root Length (%)			
	NaCl (mM)		ABA (µM)	
	125	150	5	10
Col-0	64.6 ± 7.2	77.2 ± 4.3	19.4 ± 7.2	29.0 ± 4.5
*mterf6-5*	55.8 ± 6.0 **	63.5 ± 4.6 **	9.4 ± 13.1 **	23.2 ± 14.7

The values correspond to the root length inhibition percentages of the plants transferred 5 DAS to the media supplemented with either 125 or 150 mM of NaCl or 5 or 10 µM of ABA, which refers to those of plants of the same genotype, which were transferred to the non-supplemented media. Eight days after transfer (13 DAS), the main root length was determined per plant to evaluate their tolerance to these stress conditions (see Materials and Methods). Each value is the mean ± SD of the main root length of at least 20 plants per genotype and condition. The values significantly differed from the Col-0 at ** *p* < 0.01 according to a Student’s *t*-test.

## Supplementary Materials

Supplementary materials can be found at http://www.mdpi.com/1422-0067/19/8/2388/s1.

## Abbreviations

mTERF	mitochondrial transcription termination factor
SHOT1	SUPPRESSOR OF HOT1-4 1
OGE	organellar gene expression
SOLDAT10	SINGLET OXYGEN-LINKED DEATH ACTIVATOR10
*MOC1*	*mterf-like gene of Chlamydomonas1*
ABA	abscisic acid
DAS	days after stratification
*mda1*	*mterf defective in Arabidopsis1*
*RD29A*	*RESPONSIVE TO DESICCATION29A*
