# Supplementary material for: The Characterization of Arabidopsis mterf6 Mutants Reveals a New Role for mTERF6 in Tolerance to Abiotic Stress
Source: Int J Mol Sci. 2018 Aug 14;19(8):2388. doi: 10.3390/ijms19082388 (PMC6121570; doi:10.3390/ijms19082388)
Supplement: Supplementary file 1 [file ijms-19-02388-s001.zip › ijms-337619 supplementary/ijms-337619 supplementary figures.docx]

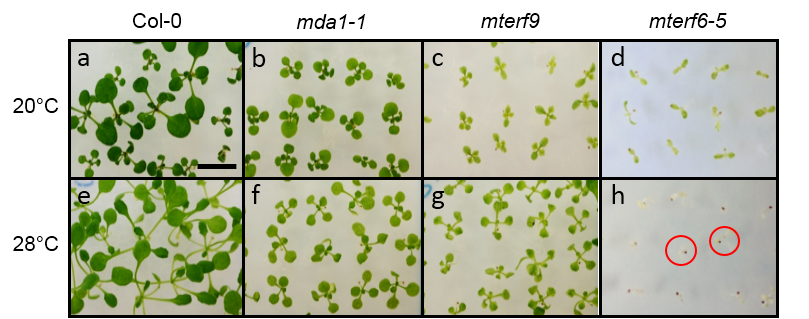


**Figure S1.** Response of the wild-type Col-0 and the *mterf6-5* mutant to moderate heat stress. Mutants *mda1-1* and *mterf9* were included as controls. The pictures correspond to representative plants for the (**a**,**e**) Col-0, (**b**,**f**) *mda1-1*, (**c**,**g**) *mterf9* and (**d**,**h**) *mterf6-5* mutants, grown at (**a**–**d**) 20 °C or (**e**–**h**) 28 °C. Circles highlight *mterf6-5* albino and arrested seedlings. Photographs were taken 13 DAS. Bar = 1 mm.


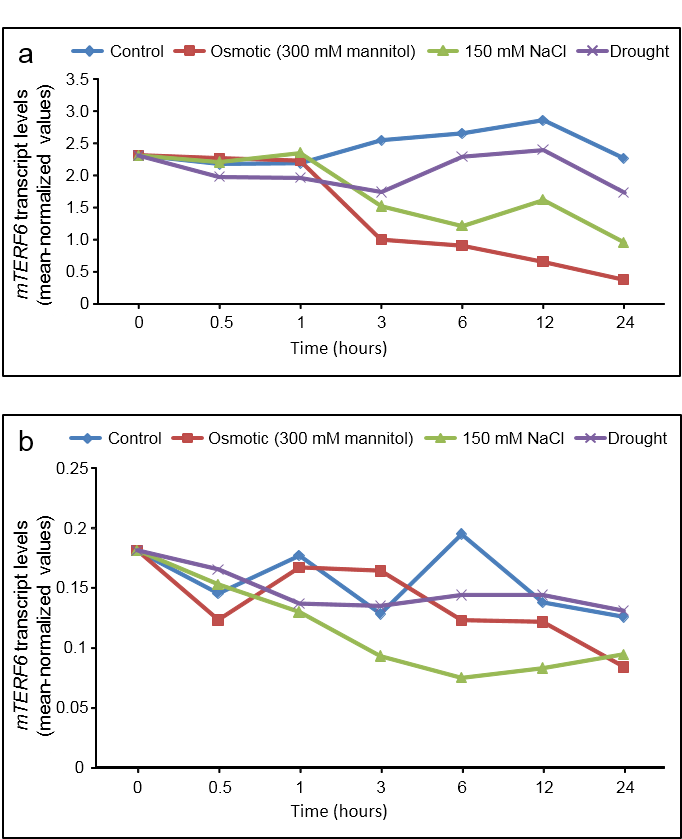


**Figure S2.** Transcript levels of the *mTERF6* gene in the wild-type plants’ response to different abiotic stress treatments. The graphs correspond to the normalised expression levels of *mTERF6* in green seedling parts (**a**) and roots (**b**) of the wild-type plants under salt (150 mM of NaCl), osmotic (300 mM of mannitol) and drought conditions. Data were obtained from the AtGenExpress Visualization Tool (http://jsp.weigelworld.org/expviz/expviz.jsp) and plotted as graphs.
