# Supplementary material for: The Characterization of Arabidopsis mterf6 Mutants Reveals a New Role for mTERF6 in Tolerance to Abiotic Stress
Source: Int J Mol Sci. 2018 Aug 14;19(8):2388. doi: 10.3390/ijms19082388 (PMC6121570; doi:10.3390/ijms19082388)
Supplement: Supplementary file 1 [file ijms-19-02388-s001.zip › ijms-337619 supplementary/Table S1 R2.docx]

**Table S1.** Germination of the wild-type Col-0 and *mterf6-5* mutant seeds on 200 mM of NaCl.

| DAS^1^ | NaCl (mM) | | | |
| --- | --- | --- | --- | --- |
|  | 0 | | 200 | |
|  | Col-0 | *mterf6-5* | Col-0 | *mterf6-5* |
| 3 | 100±0.0 | 95.0±0.0 | 0.0±0.0 | 0.0±0.0 |
| 4 | 100±0.0 | 98.0±0.0 | 51.0±4.2 | 1.0±1.4 |
| 5 | 100±0.0 | 99.0±0.0 | 98.0±0.0 | 12.1±2.8 |
| 7 | 100±0.0 | 99.0±0.0 | 99.0±1.4 | 64.6±0.0 |
| 10 | 100±0.0 | 99.0±0.0 | 99.0±1.4 | 80.8±0.0 |
| 13 | 100±0.0 | 99.0±0.0 | 99.0±1.4 | 83.8±1.4 |

Each value corresponds to the mean±the standard deviation (SD) of the percentage of germination of two-three replicates of 50-100 seeds each. The results shown correspond to a representative experiment of two independent experiments. ^1^DAS: days after stratification.
