# Supplementary material for: The Characterization of Arabidopsis mterf6 Mutants Reveals a New Role for mTERF6 in Tolerance to Abiotic Stress
Source: Int J Mol Sci. 2018 Aug 14;19(8):2388. doi: 10.3390/ijms19082388 (PMC6121570; doi:10.3390/ijms19082388)
Supplement: Supplementary file 1 [file ijms-19-02388-s001.zip › ijms-337619 supplementary/Table S3 R2.docx]

**Table S3.** Germination and seedling establishment of the wild-type Col-0 and mterf6-5 mutant seeds on 6 µM of ABA.

| DAS^2^ | Germination^1^ | | Seedling establishment | | | |
| --- | --- | --- | --- | --- | --- | --- |
|  | 6 µM ABA | | 0 µM ABA | | 6 µM ABA | |
|  | Col-0 | *mterf6-5* | Col-0 | *mterf6-5* | Col-0 | *mterf6-5* |
| 3 | 0.0±0.0 | 0.0±0.0 | 0.0±0.0 | 0.0±0.0 | 0.0±0.0 | 0.0±0.0 |
| 4 | 2.0±0.0 | 2.0±0.0 | 49.0±1.4 | 0.0±0.0 | 0.0±0.0 | 0.0±0.0 |
| 5 | 3.0±1.4 | 6.0±2.8 | 99.0±1.4 | 33.0±1.4 | 0.0±0.0 | 0.0±0.0 |
| 6 | 44.0±5.6 | 22.0±0.0 | 100.0±0.0 | 57.0±7.0 | 0.0±0.0 | 0.0±0.0 |
| 7 | 62.0±5.6 | 36.0±2.8 | 100.0±0.0 | 90.0±11.3 | 0.0±0.0 | 0.0±0.0 |
| 10 | 99.0±1.4 | 60.0±2.8 | 100.0±0.0 | 98.0±2.8 | 18.0±2.8 | 0.0±0.0 |
| 13 | 100.0±0.0 | 82.0±0.0 | 100.0±0.0 | 100.0±0.0 | 42.0±0.0 | 0.0±0.0 |

Each value corresponds to the mean± standard deviation (SD) of the percentage of germination of two-three replicates of 50-100 seeds each. The results shown correspond to a representative experiment of two independent experiments. ^1^Both strains germinated 100% in the control (non-supplemented) growth medium from 3 to 13 DAS. ^2^DAS: days after stratification.
