# Supplementary material for: The Characterization of Arabidopsis mterf6 Mutants Reveals a New Role for mTERF6 in Tolerance to Abiotic Stress
Source: Int J Mol Sci. 2018 Aug 14;19(8):2388. doi: 10.3390/ijms19082388 (PMC6121570; doi:10.3390/ijms19082388)
Supplement: Supplementary file 1 [file ijms-19-02388-s001.zip › ijms-337619 supplementary/Table S4 R2.docx]

**Table S4.** Primers used in this work.

| Purpose | Primer names  (forward; F/reverse; R) | Oligonucleotide sequences (5´→3´) | |
| --- | --- | --- | --- |
|  |  | Forward primer | Reverse primer |
| qRT-PCR | mTERF6 F/R(Q) | GGGACTACCTGAGTAACATTGT | CCGAGGATTCCTTCCAAGACT |
|  | RD29A-F/R(Q) | ATGCACCAGGCGTAACAGG | TCTTGTACTGGTACAGATT |
|  | ACTIN2 F/R(Q) | GCACCCTGTTCTTCTTACCG | AACCCTCGTAGATTGGCACA |
